# Heritability and genetic correlation estimates of semen production traits with litter traits and pork production traits in purebred Duroc pigs

**DOI:** 10.1093/jas/skac055

**Published:** 2022-02-24

**Authors:** Shinichiro Ogawa, Makoto Kimata, Masamitsu Tomiyama, Masahiro Satoh

**Affiliations:** 1 Graduate School of Agricultural Science, Tohoku University, Sendai, Miyagi 980-8572, Japan; 2 CIMCO Corporation, Koto-ku, Tokyo 136-0071, Japan

**Keywords:** Duroc pigs, genetic parameter estimation, litter traits, pork production traits, semen production traits

## Abstract

We estimated heritabilities of semen production traits and their genetic correlations with litter traits and pork production traits in purebred Duroc pigs. Semen production traits were semen volume, sperm concentration, proportion of morphologically normal sperms, total number of sperm, and total number of morphologically normal sperm. Litter traits at farrowing were total number born, number born alive, number stillborn, total litter weight at birth, mean litter weight at birth, and piglet survival rate at birth. Litter traits at weaning were litter size at weaning, total litter weight at weaning, mean litter weight at weaning, and piglet survival rate from birth to weaning. Pork production traits were average daily gain, backfat thickness, and loin muscle area. We analyzed 45,913 semen collection records of 896 boars, 6,950 farrowing performance records of 1,400 sows, 2,237 weaning performance records of 586 sows, and individual growth performance records of 9,550 animals measured at approximately 5 mo of age. Heritabilities were estimated using a single-trait animal model. Genetic correlations were estimated using a 2-trait animal model. Estimated heritabilities of semen production traits ranged from 0.20 for sperm concentration to 0.29 for semen volume and were equal to or higher than those of litter traits, ranging from 0.06 for number stillborn and piglet survival rate at birth to 0.25 for mean litter weight at birth, but lower than those of pork production traits, ranging from 0.50 for average daily gain to 0.63 for backfat thickness. In many cases, the absolute values of estimated genetic correlations between semen production traits and other traits were smaller than 0.3. These estimated genetic parameters provide useful information for establishing a comprehensive pig breeding scheme.

## Lay Summary

Genetic parameters of 5 semen production traits, 10 litter traits, and 3 pork production traits in purebred Duroc pigs was estimated. Heritabilities of semen production traits ranged from 0.20 for sperm concentration to 0.29 for semen volume and were equal to or higher than those of litter traits, ranging from 0.06 for number stillborn and piglet survival rate at birth to 0.25 for mean litter weight at birth, but lower than those of pork production traits, ranging from 0.50 for average daily gain to 0.63 for backfat thickness. In many cases, the absolute values of genetic correlations between semen production traits and other traits were smaller than 0.3. These estimated genetic parameters provide useful information for establishing a comprehensive pig breeding scheme.

## Teaser

Genetic parameters of 5 semen production traits, 10 litter traits, and 3 pork production traits in purebred Duroc pigs were estimated, providing useful information for establishing a comprehensive pig breeding scheme.

## Introduction

The Duroc breed is a terminal sire breed widely used in crossbreeding for modern pork production. In Japan, efforts have been made to improve pork productivity and quality in Duroc pig populations (Suzuki et al., 2005a; Ohnishi and Satoh, 2018; Yazaki et al., 2020). To achieve more efficient pork production, it is important to improve feed efficiency (Hoque et al., 2007; Ito et al., 2018; Homma et al., 2021) and reproductive efficiency, such as increasing litter size (Tomiyama et al., 2011; Konta et al., 2019; Ogawa et al., 2019b) and using artificial insemination techniques (González-Peña et al., 2015, 2016; Krupa et al., 2020). In regard to semen production traits, including semen volume (**VOL**) and total number of sperm (**NUM**) per ejaculate, previous studies have estimated genetic parameters by using a repeatability animal model (0.1–0.3; Wolf, 2010; Marques et al., 2017; Zhao et al., 2019), and moderate heritabilities were often estimated (Zak et al., 2017). Recent studies have investigated the underlying genetic mechanism (Marques et al., 2018; Gòdia et al., 2019; Mei et al., 2021). For Landrace and Large White breeds, genetic correlations of semen production traits with total number born (**TNB**), number born alive (**NBA**), number stillborn (**NSB**), and litter size at weaning (**LSW**) have been also estimated (Wolf, 2010; Brinke et al., 2020). Nevertheless, information on the genetic relationship of semen production traits with other economically important traits in pigs is still limited. Here, we estimated the heritabilities of semen production traits and their genetic correlations with litter traits and pork production traits in purebred Duroc pigs.

## Materials and Methods

### Ethics statement

Approval of the Animal Care and Use Committee was not required for this study because the data were acquired from an existing database.

### Phenotypic and Pedigree Information

The 50,646 semen collection records from the sperm-rich fraction of 966 Duroc boars collected during 2000 and 2017, 8,128 litter performance records of 1,786 sows collected during 2000 and 2018, and individual records of performance testing at about 5 mo of age of 11,806 animals collected during 2004 and 2018 were used. These data were provided by CIMCO Corporation (Tokyo, Japan), which operates great-grandparent (**GGP**) and grandparent (**GP**) farms by Specific Pathogen Free system in northern to southern parts of Japan. Sows were serviced typically three times by artificial insemination with semen collected from the same boar. Pedigree data covered 67,993 animals. Five semen production traits per ejaculate were analyzed: VOL, sperm concentration (**CON**), proportion of morphologically normal sperm (**PROP**), NUM, calculated as VOL × CON, and total number of morphologically normal sperm (**NUMN**), calculated as NUM × PROP. Six litter traits at farrowing were analyzed: TNB, NBA, NSB, total litter weight at birth (**LWB**), mean litter weight at birth (**MWB**), calculated as LWB/NBA, and piglet survival rate at birth (**SVB**), calculated as NBA/TNB. Four litter traits at weaning at 21 d after farrowing were analyzed: LSW, total litter weight (**LWW**), mean litter weight (**MWW**), calculated as LWW/ LSW, and piglet survival rate from birth to weaning (**SVW**), calculated as LSW/ NBA. Three pork production traits were analyzed: average daily gain (**ADG**), calculated as live body weight/ age at end of testing, and ultrasonically measured backfat thickness (**BF**) and loin muscle area (**LMA**) at end of testing.

For semen production traits, we first excluded semen collection records with an interval of longer than 21 d from the previous collection, and age at ejaculation of younger than 6 or older than 48 mo. Then, we extracted records with VOL and CON within the ranges of their means ± 3 SD, collecting 45,913 records of 896 boars. For litter traits, we extracted litter performance records at farrowing collected after 2001 using semen from Duroc boars and extracted those at weaning without cross-fostering, according to previous studies of Landrace and Large White pigs in the same company (Ogawa et al., 2019a, b). This gave 6,950 records at farrowing from 1,400 sows and 2,237 records at weaning from 586 sows. For pork production traits, we extracted performance testing records of 9,550 animals tested on GGP farms with live body weight between 80 and 130 kg, ADG between 300 and 1,500 × *g*, BF between 5 and 50 mm, and LMA between 15 and 60 cm^2^, according to the national swine genetic evaluation in Japan (http://www.nlbc.go.jp/kachikukairyo/iden/buta/hyoka_meat2.pdf). Table 1 lists descriptive statistics of the 18 traits studied.

**Table 1.** Descriptive statistics of the traits studied

Trait; abbreviation	Unit	No. of animals	No. of records	Mean	SD	Min	Max
Semen volume; VOL	ml	896	45,913	126.7	56.8	9	321
Sperm concentration; CON	10^8^ counts/ml	896	45,913	5.81	2.36	0.24	13.73
Proportion of morphologically normal sperm; PROP	—	896	45,913	0.89	0.05	0.10	1
Total number of sperm; NUM	10^8^ counts	896	45,913	660.3	245.6	5.7	2,753.1
Total number of morphologically normal sperm; NUMN	10^8^ counts	896	45,913	588.3	221.5	5.2	2,505.3
Total number born; TNB	Number	1,400	6,950	9.8	2.6	1	18
Number born alive; NBA	Number	1,400	6,950	9.1	2.4	1	17
Number stillborn; NSB	Number	1,400	6,950	0.7	1.0	0	8
Total litter weight at birth; LWB	kg	1,400	6,950	14.1	3.6	1.2	26.5
Mean litter weight at birth; MWB	kg	1,400	6,950	1.58	0.25	0.81	2.17
Survival rate at birth; SVB	—	1,400	6,950	0.94	0.09	0.33	1
Litter size at weaning; LSW	number	586	2,237	7.7	2.1	1	13
Total litter weight at weaning; LWW	kg	586	2,237	41.4	11.2	5	76.8
Mean litter weight at weaning; MWW	kg	586	2,237	5.54	1.12	3.10	9.48
Survival rate from birth to weaning; SVW	—	586	2,237	0.83	0.16	0.15	1
Average daily gain; ADG	g/d	9,550	9,550	704.8	64.5	519.5	921.4
Backfat thickness; BF	mm	9,550	9,550	26.1	7.1	10.3	50
Loin muscle area; LMA	cm^2^	9,550	9,550	28.4	5.0	15.0	45.1

### Numerical analysis

The following repeatability animal model was fitted to the phenotypic records for semen production and litter traits:


$$\begin{array}{l} y=Xb+Za+Wpe+e, \end{array}$$

where **y** is the vector of phenotypic records; **b** is the vector of fixed effects; **a** is the vector of breeding values; **pe** is the vector of permanent environmental effects; **e** is the vector of errors; and **X**, **Z**, and **W** are the design matrices relating **b**, **a**, and **pe**, respectively, to **y**. Fixed effects for semen production traits (Wolf and Smital, 2009a, b; Wolf, 2010) were year at ejaculation (18 levels; 2000 to 2017); month at ejaculation (12 levels; January to December); farm (6 levels; two GGP and four GP farms); age at ejaculation (43 levels: 6 to 48 mo), and interval between present and previous semen collections (13 levels: equal to or shorter than 3, 4, …, 14, and equal to or longer than 15 d). Fixed effects for litter traits (Ogawa et al., 2019a, b) were farrowing year (18 levels: 2001 to 2018), farrowing season (4 levels: spring [March to May], summer [June to August], autumn [September to November], winter [December to February]), farm (6 levels: two GGP farms and four GP farms), and dam parity (10 levels: 1st to 9th and equal to or higher than 10th).

The following animal model was fitted to the phenotypic records for pork production traits:


$$\begin{array}{l} y=Xb+Za+e. \end{array}$$

Fixed effects were sex (2 levels; boar, gilt); farm (2 levels; two GGP farms), birth year (13 levels; 2006 to 2018), month at end of testing (12 levels; January to December), and a linear covariate of live body weight at end of testing, similar to the statistical model used in the national swine genetic evaluation in Japan.

Heritability was estimated via single-trait analysis and genetic correlation was estimated via 2-trait analysis. We confirmed that the estimated heritabitities from 2-trait analysis were similar to those from single-trait analysis (Supplementary Table S1). When estimating the heritabilities of semen production and litter traits, the mean and (co)variance of the vectors **a**, **pe**, and **e** were assumed as follows:


$$\begin{array}{l} E\left[\begin{array}{l} a\\ pe\\ e \end{array}\right]=\left[\begin{array}{l} 0\\ 0\\ 0 \end{array}\right]and\mathrm{var}\left[\begin{array}{l} a\\ pe\\ e \end{array}\right]=\left[\begin{array}{lll} A\sigma_{a}^{2} & 0 & 0\\ 0 & I\sigma_{pe}^{2} & 0\\ 0 & 0 & I\sigma_{e}^{2} \end{array}\right], \end{array}$$

where $\sigma_{a}^{2}$ is the additive genetic variance; $\sigma_{pe}^{2}$ is the permanent environmental variance; $\sigma_{e}^{2}$ is the error variance; ***A*** is the additive genetic relationship matrix; and ***I*** is the identity matrix. When estimating the heritabilities of pork production traits, the mean and (co)variance of **a** and **e** were assumed as


$$\begin{array}{l} E\left[\begin{array}{l} a\\ e \end{array}\right]=\left[\begin{array}{l} 0\\ 0 \end{array}\right]and\mathrm{var}\left[\begin{array}{l} a\\ e \end{array}\right]=\left[\begin{array}{ll} A\sigma_{a}^{2} & 0\\ 0 & I\sigma_{e}^{2} \end{array}\right]. \end{array}$$

When estimating the genetic correlation between the 2 semen production traits and that between the 2litter traits, the mean and (co)variance of **a**, **pe**, and **e** were assumed as follows:


$$\begin{array}{l} E\left[\begin{array}{l} a_{1}\\ a_{2}\\ pe_{1}\\ pe_{2}\\ e_{1}\\ e_{2} \end{array}\right]=\left[\begin{array}{l} 0\\ 0\\ 0\\ 0\\ 0\\ 0 \end{array}\right]and\mathrm{var}\left[\begin{array}{l} a_{1}\\ a_{2}\\ pe_{1}\\ pe_{2}\\ e_{1}\\ e_{2} \end{array}\right]=\left[\begin{array}{llllll} A\sigma_{a1}^{2} & A\sigma_{a12} & 0 & 0 & 0 & 0\\ A\sigma_{a12} & A\sigma_{a2}^{2} & 0 & 0 & 0 & 0\\ 0 & 0 & I\sigma_{pe1}^{2} & I\sigma_{pe12} & 0 & 0\\ 0 & 0 & I\sigma_{pe12} & I\sigma_{pe2}^{2} & 0 & 0\\ 0 & 0 & 0 & 0 & I\sigma_{e1}^{2} & I\sigma_{e12}\\ 0 & 0 & 0 & 0 & I\sigma_{e12} & I\sigma_{e2}^{2} \end{array}\right], \end{array}$$

where $\sigma_{a12}$ is the additive genetic covariance; $\sigma_{pe12}$ is the permanent environmental covariance; $\sigma_{e12}$ is the error covariance; and subscripts correspond to traits. When estimating the genetic correlation between the two pork production traits, the mean and (co)variance of **a** and **e** were assumed as


$$\begin{array}{l} E\left[\begin{array}{l} a_{1}\\ a_{2}\\ e_{1}\\ e_{2} \end{array}\right]=\left[\begin{array}{l} 0\\ 0\\ 0\\ 0 \end{array}\right]and\mathrm{var}\left[\begin{array}{l} a_{1}\\ a_{2}\\ e_{1}\\ e_{2} \end{array}\right]=\left[\begin{array}{llll} A\sigma_{a1}^{2} & A\sigma_{a12} & 0 & 0\\ A\sigma_{a12} & A\sigma_{a2}^{2} & 0 & 0\\ 0 & 0 & I\sigma_{e1}^{2} & I\sigma_{e12}\\ 0 & 0 & I\sigma_{e12} & I\sigma_{e2}^{2} \end{array}\right]. \end{array}$$

When estimating the genetic correlation between semen production and litter traits, the mean and (co)variance of **a**, **pe**, and **e** were assumed (Wolf, 2010), as


$$\begin{array}{l} E\left[\begin{array}{l} a_{1}\\ a_{2}\\ pe_{1}\\ pe_{2}\\ e_{1}\\ e_{2} \end{array}\right]=\left[\begin{array}{l} 0\\ 0\\ 0\\ 0\\ 0\\ 0 \end{array}\right]and\mathrm{var}\left[\begin{array}{l} a_{1}\\ a_{2}\\ pe_{1}\\ pe_{2}\\ e_{1}\\ e_{2} \end{array}\right]=\left[\begin{array}{llllll} A\sigma_{a1}^{2} & A\sigma_{a12} & 0 & 0 & 0 & 0\\ A\sigma_{a12} & A\sigma_{a2}^{2} & 0 & 0 & 0 & 0\\ 0 & 0 & I\sigma_{pe1}^{2} & 0 & 0 & 0\\ 0 & 0 & 0 & I\sigma_{pe2}^{2} & 0 & 0\\ 0 & 0 & 0 & 0 & I\sigma_{e1}^{2} & 0\\ 0 & 0 & 0 & 0 & 0 & I\sigma_{e2}^{2} \end{array}\right]. \end{array}$$

When estimating the genetic correlations of pork production traits with semen production traits and litter traits, the mean and (co)variance of **a**, **pe**, and **e** were assumed (Wolf et al., 2005; Konta et al., 2019; Ogawa et al., 2020) as


$$\begin{array}{l} E\left[\begin{array}{l} a_{1}\\ a_{2}\\ pe_{1}\\ e_{1}\\ e_{2} \end{array}\right]=\left[\begin{array}{l} 0\\ 0\\ 0\\ 0\\ 0 \end{array}\right]and\mathrm{var}\left[\begin{array}{l} a_{1}\\ a_{2}\\ pe_{1}\\ e_{1}\\ e_{2} \end{array}\right]=\left[\begin{array}{lllll} A\sigma_{a1}^{2} & A\sigma_{a12} & 0 & 0 & 0\\ A\sigma_{a12} & A\sigma_{a2}^{2} & 0 & 0 & 0\\ 0 & 0 & I\sigma_{pe1}^{2} & 0 & 0\\ 0 & 0 & 0 & I\sigma_{e1}^{2} & 0\\ 0 & 0 & 0 & 0 & I\sigma_{e2}^{2} \end{array}\right]. \end{array}$$

Variance components were estimated using AIREMLF90 software (Misztal et al., 2002). SEs were obtained according to Klei and Tsuruta (2008). Variance component estimation was stopped when at least one of the following conditions was satisfied:


$$\begin{array}{l} \frac{\sum_{i=1}^{n}{\left({\hat{\theta }}_{i,k}-{\hat{\theta }}_{i,k-1}\right)}^{2}}{\sum_{i=1}^{n}{\hat{\theta }}_{i,k}^{2}}<10^{-20}or\frac{\sum_{i=1}^{n}\left|{\hat{\theta }}_{i,k}-{\hat{\theta }}_{i,k-1}\right|}{n}<10^{-10} \end{array}$$

where ${\hat{\theta }}_{i,k}$ is the estimated value of parameter *i* in iteration *k*; and *n* is the number of parameters to be estimated.

Selection accuracies for VOL and NBA based on selection candidate’s own repeated phenotypic records were calculated using the estimated genetic parameters as:


$$\begin{array}{l} \sqrt{\frac{mh^{2}}{1+\left(m-1\right)rep^{2}}}\underset{m\to +\infty }{\to }\;\sqrt{\frac{h^{2}}{rep^{2}}}, \end{array}$$

where *h*^2^ is the heritability, rep^2^ is the repeatability, and *m* is the number of records. Here, we used genetic parameters estimated by single-trait analysis (Table 2) as true values. Then, we assumed that that age at first record collection was 8 mo, or 32 wk in this study, for VOL, and 1 yr, or 52 wk, for NBA, and that the interval between consecutive record collections was 1 wk for VOL and 23 wk for NBA. Under these assumptions, the age of a selection candidate when the number of own phenotypic records just reaches *m* can be expressed as 31 + *m* weeks for VOL and 29 + 23 *m* weeks for NBA. Next, selection accuracy for VOL was calculated by decreasing the values of permanent environmental and error variances, $\sigma_{pe}^{2}$ and $\sigma_{e}^{2}$. The value of $\sigma_{pe}^{2}$ (719.8) was decreased to 75% (to 539.9) and 50% (to 359.9) while keeping other variances the same. The value of $\sigma_{e}^{2}$ (1,807.8) was decreased by subtracting 25% and 50% of $\sigma_{pe}^{2}$ (to 1,627.8 and 1,447.9, respectively).

**Table 2.** Genetic parameters estimated by single-trait analysis

Trait^1^	Additive genetic variance		Permanent environmental variance		Error variance		Heritability (*h*^2^)	Repeatability (rep^2^)	*h*^2^ ÷ rep^2^
	Estimate	SE	Estimate	SE	Estimate	SE			
VOL	1,025.1	177.1	719.8	110.4	1,807.8	12.1	0.29	0.49	0.59
CON	1.68	0.25	0.76	0.15	3.54	0.02	0.28	0.41	0.69
PROP	2.26 × 10^4^	0.43 × 10^4^	3.00 × 10^4^	0.32 × 10^4^	6.21 × 10^4^	0.04 × 10^4^	0.20	0.46	0.43
NUM	11.11 × 10^3^	2.04 × 10^3^	10.50 × 10^3^	1.36 × 10^3^	26.83 × 10^3^	0.18 × 10^3^	0.23	0.45	0.51
NUMN	8.73 × 10^3^	1.66 × 10^3^	9.02 × 10^3^	1.12 × 10^3^	21.67 × 10^3^	0.14 × 10^3^	0.22	0.45	0.49
TNB	0.76	0.15	0.69	0.12	4.73	0.09	0.12	0.23	0.53
NBA	0.62	0.12	0.57	0.10	4.25	0.08	0.11	0.22	0.52
NSB	0.05	0.01	0.03	0.01	0.80	0.01	0.06	0.09	0.67
LWB	1.98	0.35	1.62	0.26	7.74	0.15	0.17	0.32	0.55
MWB	1.51 × 10^2^	0.20 × 10^2^	0.17 × 10^2^	0.12 × 10^2^	4.48 × 10^2^	0.08 × 10^2^	0.25	0.27	0.90
SVB	0.45 × 10^3^	0.12 × 10^3^	0.16 × 10^3^	0.10 × 10^3^	7.07 × 10^3^	0.13 × 10^3^	0.06	0.08	0.74
LSW	0.39	0.14	0.36	0.13	3.25	0.11	0.10	0.19	0.52
LWW	18.39	5.32	13.47	4.21	74.37	2.59	0.17	0.30	0.58
MWW	0.10	0.04	0.07	0.03	0.96	0.33	0.09	0.14	0.59
SVW	0.15 × 10^2^	0.06 × 10^2^	0.12 × 10^2^	0.06 × 10^2^	1.92 × 10^2^	0.07 × 10^2^	0.07	0.12	0.55
ADG	69.0	4.1	—	—	70.3	2.3	0.50	—	—
BF	12.6	0.7	—	—	7.3	0.4	0.63	—	—
LMA	5.82	0.33	—	—	4.43	0.18	0.57	—	—

See Table 1 for abbreviations of trait names.

## Results and Discussion

### Estimated heritabilities and repeatabilities

Estimated heritabilities of semen production traits ranged from 0.20 for PROP to 0.29 for VOL, equal to or higher than those of litter traits (ranging from 0.06 for NSB and SVB to 0.25 for MWB), but lower than those of pork production traits (from 0.50 for ADG to 0.63 for BF; Table 2). The heritabilities of several semen production traits, estimated using a repeatability model (0.1 to 0.3) here and elsewhere (Wolf, 2010; Buranawit and Imboonta, 2016; Li et al., 2019; Brinke et al., 2020), were higher than those of TNB, NBA, NSB, and LSW in Landrace and Large White populations. Our estimated heritabilities of litter traits agreed with those in studies of Landrace and Large White populations in the same company and different Duroc populations in Japan (Ohnishi and Satoh, 2014; Ogawa et al., 2019b, 2020). Heritabilities of pork production traits may be overestimated if common environmental effects such as maternal, pen, littermate, and pen-mate effects are ignored (Willham, 1963; Ellen et al., 2007; Ogawa et al., 2021). Furthermore, the estimated phenotypic variance for ADG seems small. In this study, it is likely that the variance of phenotypic records for ADG reflects the variability in body weight more than that in age at end of testing. In fact, when we used the model ignoring the effect of body weight, estimated values of additive genetic and error variances increased from 69.0 to 1,653.5 and from 70.3 to 1,692.9, respectively.

Estimated repeatabilities for semen production traits ranged from 0.41 for CON to 0.49 for VOL, and they were higher than those of litter traits (from 0.09 for NSB to 0.32 for LWB). Estimated repeatabilities for litter traits agreed with those of Landrace and Large White populations of the same company and in another Duroc population in Japan (Ogawa et al., 2019b, 2020). Wolf (2010) estimated proportions of permanent environmental variance to the phenotypic variance of several semen production traits to be higher than those of TNB, NBA, and LSW in Landrace and Large White populations. Ratios of estimated heritability to estimated repeatability ranged from 0.43 for PROP to 0.90 for MWB, comparable to the estimated heritabilities of pork production traits.

### Estimated genetic correlations

The absolute values of the genetic correlation between pairs of semen production traits were more than twice their respective SEs, except for PROP and NUM and PROP and NUMN (Table 3). The estimated genetic correlation between NUM and NUMN was almost 1, possibly due to low contribution of PROP to NUMN in this study. As reported in previous studies (Wolf, 2010; Buranawit and Imboonta, 2016; Brinke et al., 2020), genetic correlations of VOL were positive with NUM and NUMN and negative with CON, and those of CON were positive with NUM and NUMN. The genetic correlation between CON and PROP was −0.33 here, whereas between CON and percentage of deformed or abnormal sperm cells was 0.13 in Landrace and −0.14 in Large White (Wolf, 2010), and −0.34 in Duroc, 0.08 in Landrace, and −0.04 in Yorkshire (Li et al., 2019). Previous studies have estimated different genetic correlations for some pairs of semen production traits among breeds and lines (Wolf, 2010; Marques et al., 2017; Li et al. 2019). Semen production records could be mainly collected from sires after strict selection based on a particular breeding objective, which may be hampering the consistency of results across studies.

**Table 3.** Estimated genetic correlations (below diagonal) and their SEs (above diagonal)

Trait^1^	Trait																	
	VOL	CON	PROP	NUM	NUMN	TNB	NBA	NSB	LWB	MWB	SVB	LSW	LWW	MWW	SVW	ADG	BF	LMA
VOL	-	0.07	0.12	0.10	0.10	0.15	0.15	0.17	0.13	0.12	0.17	0.20	0.18	0.22	0.22	0.08	0.08	0.07
CON	−0.66^**^	-	0.11	0.11	0.11	0.13	0.13	0.15	0.12	0.11	0.16	0.19	0.16	0.20	0.20	0.07	0.07	0.07
PROP	0.18^*^	−0.33^**^	-	0.13	0.13	0.14	0.15	0.16	0.14	0.12	0.16	0.20	0.18	0.21	0.22	0.09	0.09	0.08
NUM	0.48^**^	0.27^**^	−0.12	-	0.00	0.14	0.15	0.16	0.14	0.12	0.17	0.21	0.18	0.23	0.22	0.08	0.08	0.08
NUMN	0.51^**^	0.23^**^	−0.02	0.99^**^	-	0.15	0.15	0.17	0.14	0.12	0.17	0.21	0.18	0.23	0.22	0.08	0.08	0.08
TNB	0.09^*^	−0.26^*^	0.12	−0.19^*^	−0.18^*^	-	0.01	0.13	0.06	0.11	0.15	0.09	0.13	0.18	0.18	0.09	0.09	0.09
NBA	0.12	−0.20^*^	0.18^*^	−0.12	−0.09	0.96^**^	-	0.16	0.07	0.11	0.16	0.07	0.12	0.18	0.19	0.09	0.09	0.09
NSB	-0.10	-0.34^**^	-0.17^*^	-0.39^**^	-0.44^**^	0.46^**^	0.21^*^	-	0.13	0.12	0.02	0.23	0.19	0.22	0.21	0.11	0.10	0.10
LWB	0.25^*^	-0.31^**^	0.18^*^	-0.05	-0.03	0.71^**^	0.65^**^	0.48^**^	-	0.08	0.14	0.10	0.08	0.18	0.20	0.08	0.08	0.08
MWB	0.16^*^	-0.14^*^	0.04	0.08	0.09	-0.12^*^	-0.25^**^	0.38^**^	0.57^**^	-	0.11	0.16	0.12	0.10	0.16	0.07	0.07	0.07
SVB	0.19^*^	0.31^*^	0.22^*^	0.40^**^	0.46^**^	-0.20^*^	0.06	-0.96^**^	-0.27^*^	-0.43^**^	-	0.20	0.19	0.22	*NA*	0.11	0.10	0.11
LSW	0.25^*^	-0.08	0.26^*^	0.12	0.17	0.77^**^	0.85^**^	-0.14	0.71^**^	0.07	0.52^**^	-	0.09	0.26	0.23	0.14	0.13	0.13
LWW	0.22^*^	-0.09	0.11	0.14	0.16	0.50^**^	0.54^**^	0.12	0.79^**^	0.51^**^	0.20^*^	0.80^**^	-	0.18	0.19	0.11	0.10	0.11
MWW	0.03	0.09	-0.11	0.23^*^	0.22	-0.17	-0.26^*^	0.36^*^	0.41^**^	0.72^**^	-0.36^*^	-0.06	0.56^*^	-	0.28	0.15	0.13	0.15
SVW	0.53^**^	0.08	-0.07	0.40^*^	0.43^*^	-0.37^**^	-0.26^*^	-0.51^**^	0.10	0.39^**^	*NA* ^2^	0.41^*^	0.50^*^	0.11	-	0.15	0.15	0.15
ADG	-0.02	0.07^*^	-0.01	0.05	0.05	0.02	0.01	0.07	0.06	0.04	-0.04	-0.05	0.07	0.23^*^	-0.20^*^	-	0.04	0.04
BF	0.03	0.02	0.01	-0.02	-0.03	-0.04	-0.02	-0.09	-0.24^**^	-0.24^**^	0.09	-0.02	-0.26^**^	-0.56^**^	0.06	-0.03	-	0.04
LMA	0.02	-0.04	-0.12^*^	-0.12^*^	-0.14^*^	0.04	0.04	0.05	0.03	0.00	-0.05	0.18^*^	0.15^*^	-0.04	-0.03	0.05^*^	-0.16^**^	-

See Table 1 for abbreviations of trait names.

*NA*: could not be reliably estimated.

The absolute value of the estimate was greater than its SE.

The absolute value of the estimate was two times greater than its SE.

Estimated genetic correlations among litter traits in Table 3 agree with those in Landrace and Large White populations of the same company and different Japanese Duroc populations (Ohnishi and Satoh, 2014; Ogawa et al., 2019b, 2020). The genetic correlation between SVB and SVW could not be reliably estimated, because the estimated permanent environmental variance of SVW was too small, at 10^-10^. This problem may be associated with the facts that the estimated heritabilities of SVB and SVW were both low in single-trait analyses and that NBA was the numerator of SVB but the denominator of SVW. With the approximation formula extended by Ogawa and Satoh (2020) from the formula proposed by Sutherland (1965), the indirectly estimated genetic correlation between SVB and SVW was 0.34. However, the discrepancy between the estimated and the true values may be large, because the SEs of estimates used in the approximation calculation were not small.

Estimated genetic correlations among pork production traits were weak to negligible (Table 3). Previous studies of Japanese Duroc populations have reported a weak positive genetic correlation between ADG and BF and weak negative genetic correlations between ADG and LMA and between BF and LMA (Suzuki et al., 2005a; Ito et al., 2018; Ohnishi and Satoh, 2018; Yazaki et al., 2020). However, the definition of ADG in the previous studies included values within predefined ranges of body weight, such as from 30 to 105 kg (Suzuki et al., 2005a; Hoque et al., 2007; Ohnishi and Satoh, 2018; Yazaki et al., 2020) or from 30 to 100 kg (Ito et al., 2018; Homma et al., 2021).

Standard errors of genetic correlations between the five semen production traits and the ten litter traits tended to be greater, ranging from 0.11 to 0.23 (Table 3). The absolute values of estimated genetic correlations of 7 trait pairs were greater than twice their respective SEs, and those of 10 pairs were larger than 0.3. Estimated genetic correlations of semen production traits with NSB, SVB, and SVW were moderate to weak, but the reason is unknown. We estimated a genetic correlation of 0.12 between VOL and NBA, whereas Brinke et al. (2020) estimated weak negative correlations in German Landrace and Large White pigs, and Wolf’s (2010) estimates had different signs depending on breed and dam parity in Czech Landrace and Large White pigs. We estimated a genetic correlation of −0.39 between NUM and NSB, but Brinke et al. (2020) estimated positive values. Wolf (2010) estimated negative genetic correlations of NUM with TNB, NBA, and LSW, but their absolute values were often lower than twice their SEs. As discussed above, the consistency of the results seems to be low, perhaps because no animal has both semen production and litter traits (Wolf, 2010), and therefore genetic correlations could be estimated only via relatives in the pedigree.

Absolute values of estimated genetic correlations between semen production traits and pork production traits were equal to or smaller than 0.14, less than twice their SEs. Wolf (2009) also estimated weak genetic correlations between semen production traits and pork production traits and noted that selection on pork production traits would have minor effects on semen production traits. Buranawit and Imboonta (2016) found no significant genetic correlation between semen production traits and pork production traits was estimated, except for −0.52 between VOL and BF.

Absolute values of estimated genetic correlations between litter traits and pork production traits were greater than twice their respective SEs only for BF with LWB, MWB, LWW, and MWW. Previous studies estimated a weak genetic correlation between litter size traits and pork production traits in pigs (Ducos and Bidanel, 1996; Misumi et al., 2009; Ogawa et al., 2020). Solé et al. (2021a, 2021b) investigated the effects of two single nucleotide polymorphisms (**SNPs**), rs709596309 C>T of the leptin receptor (***LEPR***) gene and rs80912566 T>C of the stearoyl-CoA desaturase (***SCD***) gene, on piglet weight at weaning in Duroc pigs. The SNP of *LEPR* is thought to affect BF in Duroc pigs (Óvilo et al., 2005; Uemoto et al., 2012; Ros-Freixedes et al., 2016), and may be in part responsible for the negative genetic correlation between BF and MWW here (−0.56).

### Selection accuracy

**Figure 1.**
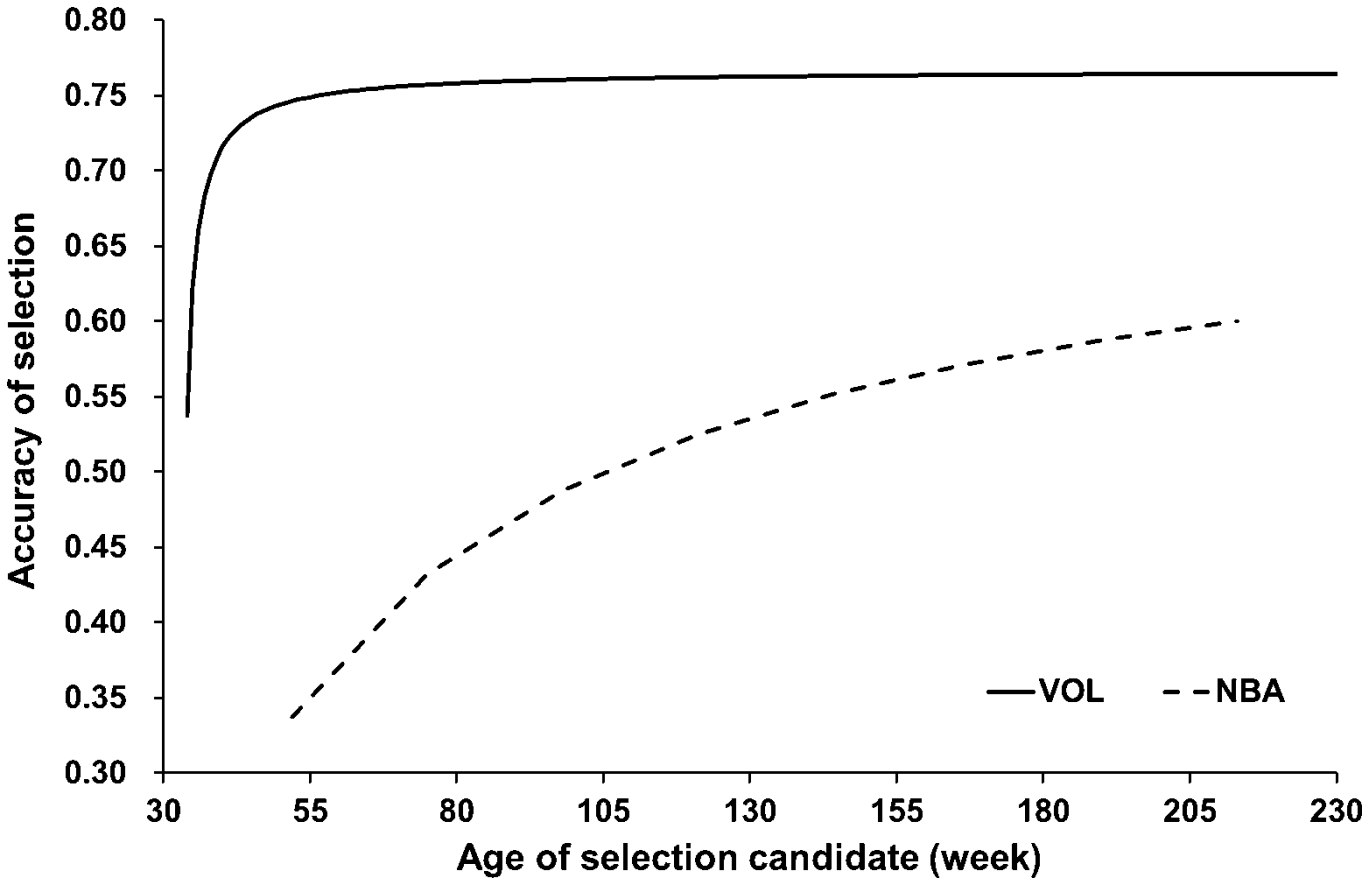
Accuracy of selection for semen volume (VOL) and number born alive (NBA) .

Selection accuracies for VOL were always greater than those for NBA (Figure 1), because the estimated heritability of VOL was higher than that of NBA (Table 2), and more records can be quickly collected for VOL than for NBA. Selection accuracy for VOL approached an upper limit, that is, close to the positive square root of heritability divided by repeatability (0.77), by about 2 years (105 wk) of age, so the contribution of collecting further records to the increase in accuracy seems to be minimal (Satoh, 2017). Thus, effective selection for semen production traits may be possible at an earlier stage than for litter traits. It is required to keep collecting semen from the same boar to increase the number of repeated records. However, it is not practical to continue to collect semen from boars not selected as sires, and the selection intensity is often higher in males than in females. Therefore, collecting semen production records from collateral relatives may be difficult, and records from direct-line relatives such as fathers of the selection candidates could be more important (Satoh, 2017).

**Figure 2.**
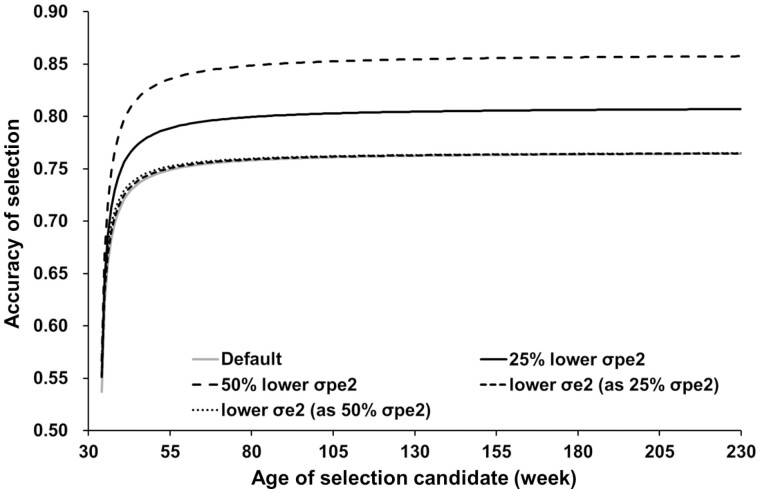
Comparison of selection accuracy for semen volume with differing permanent environmental and error variances ($\sigma_{pe}^{2}$ and$\sigma_{e}^{2}$, respectively). “Default” used the estimated values in Table 2 as true genetic parameters for calculating selection accuracy; “25% lower $\sigma_{pe}^{2}$” and “50% lower $\sigma_{pe}^{2}$” decreased the values of $\sigma_{pe}^{2}$ to 75% and 50%, respectively, of its true value; “lower $\sigma_{e}^{2}$ (as 25%$\sigma_{pe}^{2}$)” and “lower $\sigma_{e}^{2}$ (as 50%$\sigma_{pe}^{2}$)” decreased the values of $\sigma_{e}^{2}$ by subtracting 25% and 50%, respectively, of the true value of $\sigma_{pe}^{2}$.

As expected, selection accuracy increased as $\sigma_{pe}^{2}$ (more so) and $\sigma_{e}^{2}$ (less so) decreased (Figure 2) although the phenotypic variance was the same in both cases. Furthermore, as $\sigma_{pe}^{2}$ decreased, the difference in selection accuracy from that calculated with default settings at *m* records increased with *m*, whereas as $\sigma_{e}^{2}$ decreased, the difference was smaller and approached 0. Thus, adding effects so as to decrease permanent environmental variance may effectively increase the accuracy of breeding value prediction. Several studies have estimated a lower permanent environmental variance when adding non-additive genetic effects, such as dominance and epistatic effects, into repeatability model (Nagy et al., 2013; Aliloo et al., 2016; Aliloo et al., 2017; Vitezica et al., 2018). However, the possibility of more accurate breeding value prediction by adding these effects (Varona et al., 2018) may be limited, because introducing those effects brings additional covariances among individuals, as well as the difficulty in accurate estimation of non-additive genetic effects and the possible confounding problem observed in various cases, including ones using genome-wide SNP markers (Hill et al., 2008; Lee et al., 2010; Vitezica et al., 2013; Nagy et al., 2014; Bolormaa et al., 2015; Aliloo et al., 2016; Aliloo et al., 2017; Raidan et al., 2018; Joshi et al., 2020; Onogi et al., 2021).

### Future perspectives

We estimated genetic parameters of semen production traits, litter traits, and pork production traits in purebred Duroc pigs. As far as we know, no such comprehensive range of genetic parameters has previously been estimated in pigs. Therefore, the results could provide novel information useful in determining a comprehensive breeding plan for future pork production. For example, when there is an antagonistic genetic correlation between traits to be improved and litter size traits, selection intensity and selection accuracy in sib-testing may be decreased through a lower number of littermates. This could be more serious for traits relating to pork quality measured after slaughter, including intramuscular fat, fatty acid composition, and pork moisture and texture (Suzuki et al., 2005a, b, 2006). In this study, however, strong genetic correlations between litter size traits, such as NBA and LSW, and semen production and pork production traits were not estimated.

We used a repeatability model to estimate genetic parameters of semen production traits. Oh and See (2008) and Strathe et al. (2013) used multiple-trait and random-regression models, both considering the difference in age at ejaculation. It is well known that pork production can be affected by seasonal variations in the external environment, including photoperiod and temperature (Zumbach et al., 2008; Zasiadczyk et al., 2015; Sevillano et al., 2016). A detailed study of the interaction between genetics and environment (age at ejaculation and temperature) may provide useful information for breeding to resist aging and improve heat tolerance. Previous studies estimated genetic parameters in pigs and cattle using transformed semen production records (Marques et al., 2017; Hagiya et al., 2018; Rostellato et al., 2021). Therefore, it may be necessary to investigate the effect of data transformation on the performance of the analysis, as done by Zoda et al. (2021) for superovulatory response traits in Japanese Black cattle. On the other hand, for ratio-defined traits such as PROP and MWB, theoretical investigations and computer simulations have been conducted (Iwaisaki and Wilton, 1993; Ogawa and Satoh, 2020; Yazaki et al., 2021), and the results should be interpreted with caution. The results of genetic parameter estimation for semen production traits may contain bias due to both selection at a young age and data editing (Tusell et al., 2012; Atagi et al., 2017; Pelayo et al., 2019; Olsen et al., 2021). Furthermore, genetic correlations of semen production traits may differ among breeds (Wolf, 2010; Li et al., 2019). Therefore, it is important to confirm our results by conducting similar analyses for different breeds and populations with larger data sets.

## Conclusion

We estimated the heritabilities of semen production traits and their genetic correlations with litter traits at farrowing and weaning and pork production traits in purebred Duroc pigs. Estimated heritabilities of semen production traits were similar to or higher than those of litter traits. Absolute values of estimated genetic correlations between semen production traits and other traits were <0.3 in many cases, suggesting that genetic improvement of semen production traits is possible and that selection for them is unlikely to cause immediate correlated responses in other economically important traits studied. Our results could contribute to developing a future comprehensive pig breeding scheme.

## Supplementary Material

skac055_suppl_Supplementary_Table

## Abbreviations

ADG: average daily gain
BF: backfat thickness
CON: sperm concentration
GP: grandparent
GGP: great-grandparent
LEPR: leptin receptor
LMA: loin muscle area
LSW: litter size at weaning
LWB: total litter weight at birth
LWW: total litter weight at weaning
MWB: mean litter weight at birth
MWW: mean litter weight at weaning
NBA: number born alive
NSB: number stillborn
NUM: total number of sperm
NUMN: total number of morphologically normal sperm
PROP: proportion of morphologically normal sperm
SCD: stearoyl-CoA desaturase
SNP: single nucleotide polymorphism
SVB: piglet survival rate at birth
SVW: piglet survival rate until birth to weaning
TNB: total number born
VOL: semen volume
