# Supplementary material for: Heritability and genetic correlation estimates of semen production traits with litter traits and pork production traits in purebred Duroc pigs
Source: J Anim Sci. 2022 Feb 24;100(3):skac055. doi: 10.1093/jas/skac055 (PMC9030147; doi:10.1093/jas/skac055)
Supplement: skac055_suppl_Supplementary_Table [file skac055_suppl_Supplementary_Table.docx]

**Table S1.** Heritabilities estimated by two-trait analysis

| **Trait**^1^ | **Pair trait in two-trait analysis** | | | | | | | | | | | | | | | | | |
| --- | --- | --- | --- | --- | --- | --- | --- | --- | --- | --- | --- | --- | --- | --- | --- | --- | --- | --- |
|  | **VOL** | **CON** | **PROP** | **NUM** | **NUMN** | **TNB** | **NBA** | **NSB** | **LWB** | **MWB** | **SVB** | **LSW** | **LWW** | **MWW** | **SVW** | **ADG** | **BF** | **LMA** |
| VOL | - | 0.28 | 0.29 | 0.29 | 0.29 | 0.29 | 0.29 | 0.29 | 0.29 | 0.29 | 0.29 | 0.28 | 0.28 | 0.29 | 0.28 | 0.29 | 0.29 | 0.29 |
| CON | 0.28 | - | 0.28 | 0.28 | 0.28 | 0.28 | 0.28 | 0.28 | 0.28 | 0.28 | 0.28 | 0.28 | 0.28 | 0.28 | 0.28 | 0.28 | 0.28 | 0.28 |
| PROP | 0.20 | 0.20 | - | 0.19 | 0.19 | 0.20 | 0.19 | 0.20 | 0.19 | 0.20 | 0.20 | 0.20 | 0.20 | 0.20 | 0.20 | 0.20 | 0.20 | 0.20 |
| NUM | 0.23 | 0.23 | 0.23 | - | 0.23 | 0.23 | 0.23 | 0.22 | 0.23 | 0.23 | 0.22 | 0.23 | 0.23 | 0.23 | 0.23 | 0.23 | 0.23 | 0.23 |
| NUMN | 0.22 | 0.22 | 0.22 | 0.22 | - | 0.22 | 0.22 | 0.22 | 0.22 | 0.22 | 0.21 | 0.22 | 0.22 | 0.22 | 0.22 | 0.22 | 0.22 | 0.22 |
| TNB | 0.12 | 0.13 | 0.12 | 0.12 | 0.12 | - | 0.12 | 0.12 | 0.12 | 0.12 | 0.12 | 0.12 | 0.12 | 0.12 | 0.12 | 0.12 | 0.12 | 0.12 |
| NBA | 0.11 | 0.12 | 0.11 | 0.11 | 0.11 | 0.12 | - | 0.12 | 0.11 | 0.11 | 0.12 | 0.11 | 0.11 | 0.11 | 0.11 | 0.11 | 0.11 | 0.11 |
| NSB | 0.06 | 0.06 | 0.06 | 0.06 | 0.06 | 0.06 | 0.06 | - | 0.06 | 0.06 | 0.06 | 0.06 | 0.06 | 0.06 | 0.06 | 0.06 | 0.06 | 0.06 |
| LWB | 0.18 | 0.18 | 0.18 | 0.17 | 0.17 | 0.18 | 0.18 | 0.18 | - | 0.18 | 0.18 | 0.17 | 0.17 | 0.17 | 0.17 | 0.17 | 0.17 | 0.17 |
| MWB | 0.24 | 0.24 | 0.25 | 0.24 | 0.24 | 0.25 | 0.25 | 0.25 | 0.25 | - | 0.25 | 0.25 | 0.25 | 0.25 | 0.25 | 0.25 | 0.24 | 0.25 |
| SVB | 0.06 | 0.05 | 0.06 | 0.06 | 0.06 | 0.06 | 0.06 | 0.06 | 0.06 | 0.06 | - | 0.06 | 0.06 | 0.06 | 0.06 | 0.06 | 0.06 | 0.06 |
| LSW | 0.10 | 0.10 | 0.10 | 0.10 | 0.10 | 0.10 | 0.11 | 0.10 | 0.10 | 0.10 | 0.10 | - | 0.10 | 0.10 | 0.10 | 0.10 | 0.10 | 0.10 |
| LWW | 0.17 | 0.17 | 0.17 | 0.17 | 0.17 | 0.17 | 0.17 | 0.17 | 0.18 | 0.19 | 0.17 | 0.18 | - | 0.18 | 0.17 | 0.17 | 0.18 | 0.18 |
| MWW | 0.08 | 0.08 | 0.09 | 0.08 | 0.08 | 0.09 | 0.09 | 0.09 | 0.09 | 0.11 | 0.09 | 0.09 | 0.09 | - | 0.09 | 0.09 | 0.09 | 0.08 |
| SVW | 0.07 | 0.07 | 0.07 | 0.07 | 0.07 | 0.07 | 0.07 | 0.07 | 0.07 | 0.07 | 0.07 | 0.07 | 0.07 | 0.07 | - | 0.07 | 0.07 | 0.07 |
| ADG | 0.50 | 0.50 | 0.50 | 0.50 | 0.50 | 0.50 | 0.50 | 0.49 | 0.50 | 0.50 | 0.50 | 0.50 | 0.50 | 0.50 | 0.50 | - | 0.49 | 0.49 |
| BF | 0.63 | 0.63 | 0.63 | 0.63 | 0.63 | 0.63 | 0.63 | 0.63 | 0.63 | 0.63 | 0.63 | 0.63 | 0.63 | 0.63 | 0.63 | 0.63 | - | 0.63 |
| LMA | 0.57 | 0.57 | 0.57 | 0.57 | 0.57 | 0.57 | 0.57 | 0.57 | 0.57 | 0.57 | 0.57 | 0.57 | 0.57 | 0.57 | 0.57 | 0.57 | 0.57 | - |

^1^See Table 1 for abbreviations of trait names.
